# Supplementary material for: “I Had to Rediscover Our Healthy Food”: An Indigenous Perspective on Coping with Type 2 Diabetes Mellitus
Source: Int J Environ Res Public Health. 2021 Dec 24;19(1):159. doi: 10.3390/ijerph19010159 (PMC8750381; doi:10.3390/ijerph19010159)
Supplement: Supplementary file 1 [file ijerph-19-00159-s001.zip › ijerph-1461312-supplementary.pdf]

Interview guide

Topic	Questions
Opening Question	Can you tell me your story of diabetes?
Diagnosis	How long do you cope with diabetes?<br><br>What was the process of your diagnosis like?
The cause of T2DM	In your opinion what is the cause of your diabetes?
Medications and lifestyle changes	What was the process of deciding on treatment for the diabetes?<br><br>What was your role in the decision process?<br><br>Which parts of treatment are more or less difficult for you to perform?<br><br>Do you take medications for diabetes?<br><br>If so, when and how do you take medications?<br><br>Which lifestyle habits have you changed and why?<br><br>Was there a time that you decided to stop taking medications?<br><br>How do the medications affect you?<br><br>Are there any unpleasant side effects to the medications you take?<br><br>If so, how do you cope with these side effects?
Traditional medicine	Did you consult with a traditional healer? (if so, who)<br><br>Do you take traditional/herbal medications?<br><br>If you take herbal medications together with Western medications, which helps you better?

Gender and coping with diabetes	How has diabetes influenced the work you have to do inside or outside the house?<br><br>How your role at home influences your coping with diabetes?<br><br>How has diabetes influenced your relationship with your children?<br><br>How has diabetes influenced your relationship with relatives who live with you?<br><br>How has diabetes or coping with diabetes influenced your relationship with you spouse?<br><br>How do friends or relatives help you to cope with diabetes?
Treatment accessibility	Is traditional Bedouin cuisine suitable for coping with diabetes?<br><br>Are medications and medical treatment accessible in your area of living?<br><br>Which health advice or lifestyle changes recommended for you by health professionals are possible for you to make/feasible for you?<br><br>Which lifestyle changes you would have wanted to make and still haven't or feel that you are unable to make?<br><br>If and if so when do you practice physical activity?
Interactions with healthcare professionals	How would you describe your relationships with your healthcare professionals?<br><br>Give one example to an especially good experience you had with an healthcare professional and one especially bad one you had<br><br>Do you prefer to receive medical treatment from a health professional of your own gender?
Faith and religion	How does religious faith helps you to cope with diabetes (if at all)?

Social rights	How did you learn what are the social rights you are entitled to?<br><br>Which social rights did you utilize? (for example, sick leave)<br><br>Do you feel that there are social rights which might have helped you but you're not entitled to?
Social perceptions and stigma regarding T2DM	To which people in your life you told about diabetes? When?<br><br>Have you ever felt uncomfortable telling others about diabetes?<br><br>When you visit friends/relatives do you tell them that you have diabetes so they will serve you suitable foods and drinks (sugarless)?<br><br>How do you feel regarding diabetes and your coping with it?<br><br>How have your perceptions of diabetes changed over time?<br><br>How have the perceptions of you friends and relatives regarding diabetes changed over time?<br><br>Do you encounter misconceptions or prejudices about diabetes among friends or family?
Information	What information you had about T2DM before you were diagnosed?<br><br>Did you search the internet for information?<br><br>If we could arrange for you to meet the number one specialist for T2DM, what would you have wanted to tell or ask him/her?
Changes over time	Do you experience changes over time regarding coping with diabetes?<br><br>How do you see the future in relation to coping with diabetes?<br><br>How do you see the future in relation to your health?

Message to others	How in our opinion can coping with T2DM be improved, both in general and specifically among the Bedouin community?<br><br>What message would you like to give others who cope with T2DM?<br><br>What message would you like to give physicians and healthcare professionals who treat patients with T2DM?
Final questions	Is there anything I didn't ask and you would like to tell me?
Demographics	age<br><br>gender<br><br>Place of living<br><br>religion<br><br>Persona status<br><br>Number of people living in the house<br><br>Occupation
